# Arc‐ZTE: Continuously‐Slewed Zero‐TE Imaging With Incoherent Temporal Sampling for Near‐Silent Dynamic MRI

**DOI:** 10.1002/mrm.70477

**Published:** 2026-06-16

**Authors:** Shreya Ramachandran, Tobias C. Wood, Gavin Zhang, Ali B. Syed, Shreyas S. Vasanawala, Michael Lustig

**Affiliations:** ^1^ Department of Electrical Engineering and Computer Sciences University of California Berkeley Berkeley California USA; ^2^ Department of Neuroimaging King's College London London UK; ^3^ Department of Radiology Stanford University Stanford California USA

**Keywords:** dynamic MRI, RUFIS, silent MRI, ZTE

## Abstract

**Purpose:**

To enable flexible dynamic and DCE MRI with low acoustic noise and high spatiotemporal resolution using zero echo time (ZTE) imaging.

**Methods:**

This work proposes Arc‐ZTE, a technique in which the readout gradients of ZTE are continuously‐slewed to form arcs in k‐space that are sequentially rotated to yield an incoherent trajectory. These rotations are selected via an optimization procedure that both promotes temporal k‐space coverage and penalizes gradient refocusing. Arc‐ZTE was evaluated against existing radial ZTE schemes based on metrics for acoustic noise, k‐space coverage uniformity, and sampling incoherence. Phantom and in vivo demonstrations were conducted to evaluate image quality at high temporal resolutions.

**Results:**

Arc‐ZTE achieved stable k‐space coverage and improved sampling incoherence over a range of temporal resolutions, with respect to radial ZTE schemes. Although increasing arc curvature increased its acoustic noise over standard radial ZTE (by 0.3–14.2 dB), Arc‐ZTE remained substantially quieter than conventional 3D sequences by 20–35 dB. In vivo experiments demonstrated that time‐resolved Arc‐ZTE effectively captured dynamics of respiratory motion and contrast uptake when reconstructed at 0.5 and 1.1 s/frame respectively.

**Conclusion:**

Arc‐ZTE enables flexible, time‐resolved, near‐silent dynamic MRI through incoherent temporal sampling, which allows data to be retrospectively binned into a flexible choice of temporal resolution without modifying the trajectory design. This work has the potential to significantly improve the success rates of dynamic imaging among neonates, young children, and other sound‐sensitive patients.

## 1 Introduction

Dynamic volumetric MRI requires rapid and efficient k‐space sampling to achieve high spatiotemporal resolution imaging of physiological processes, such as contrast agent uptake [1, 2, 3] and pulmonary function [4]. However, the rapid gradient switching required for typical dynamic sequences generates significant acoustic noise [5], with sound pressure levels regularly reaching 95–110 dB(A) [6]. Acoustic noise is a well‐acknowledged contributor to patient discomfort [7], particularly for neonates and young children [8], where it can induce anxiety, motion [9], and even physiological stress [10]. Although generally undesirable, patient discomfort is especially problematic for dynamic contrast‐enhanced imaging, where repeat contrast injections within a single exam are often infeasible due to dose restrictions and required wash‐out time periods [11]. Derating the gradients can reduce the acoustic noise, but it comes at the cost of reduced scan efficiency.

**FIGURE 1:**
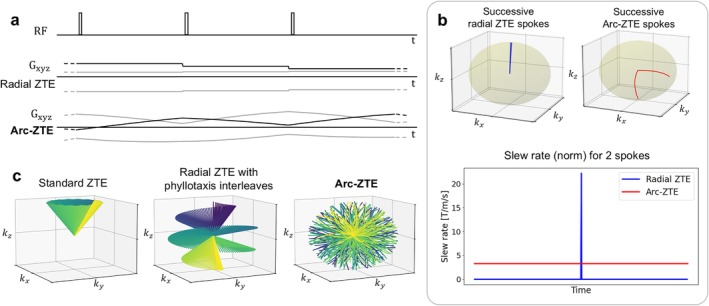
Overview of Arc‐ZTE concept. (a) Pulse sequence diagram showing 3 TRs of radial ZTE, where constant gradients are stepped by small increments between TRs, compared to Arc‐ZTE, where gradients are slewed continuously. (b) Representative example of 2 successive radial ZTE spokes (blue) and 2 successive Arc‐ZTE spokes (red) with their slew rate magnitudes. Arc‐ZTE can achieve large angular spacing between successive spokes by applying a small constant slew rate. (c) Trajectory for a continuous segment of 384 spokes for standard ZTE, radial ZTE with spiral phyllotaxis interleaves, and Arc‐ZTE. Spoke color indicates the time of its acquisition. Here, the complete standard ZTE trajectory comprises of 66 048 spokes, which corresponds to π undersampling of the theoretical Nyquist sampling for matrix size of 256.

In contrast, the zero echo time (ZTE) sequence [12], based on RUFIS [12], inherently provides both low acoustic noise and high scan efficiency [6]; hence, this sequence is an ideal candidate for near‐silent dynamic MRI. The standard ZTE sequence uses constant readout gradients that are stepped by small amounts every TR (see Figure 1a), producing closely‐spaced 3D radial spokes. This minimal gradient switching substantially reduces Lorentz forces and acoustic noise compared to conventional sequences. Moreover, since the readout gradients are not ramped down between TRs, nearly the entire TR can be utilized for data acquisition, yielding very high scan efficiencies [13]. Unlike UTE sequences, which lose efficiency from dedicated spoiler gradients [14], ZTE uses the readout gradients to spoil residual coherences from previous TRs. Since the TRs in ZTE are very short (typically < 3 ms) relative to typical T2* values, excited coherences require hundreds of TRs to completely decay; until then, these coherences can be refocused by the gradients. Hence, gradient steps between successive TRs are typically kept small to avoid unintended gradient refocusing, thereby limiting the angular distance between successive spokes (see Figure 1b).

Standard ZTE arranges radial spoke endpoints along a dense pole‐to‐pole spiral in k‐space [13, 15], but this arrangement samples only a limited section of k‐space over a short time interval. Hence, standard ZTE is typically restricted to static imaging. Alternate sampling schemes for ZTE, including AZTEK [16], phyllotaxis interleaves [17], and others [18], improve upon the standard ordering by using shorter‐duration arrangements of radial spokes that repeatedly traverse k‐space from pole to pole. However, these schemes have primarily been demonstrated at high spatial resolution for motion‐resolved imaging [16, 19], thus relying on periodic data binning into motion phases. Furthermore, the achievable temporal resolutions for these schemes are tied to the trajectory parameters; AZTEK parameters are chosen based on a pre‐recorded respiratory signal [16], and the number of spokes per phyllotaxis interleave is chosen as a divisor of the desired spokes per temporal frame [20, 21]. Other emerging ZTE works [22, 23] have curved the sequentially‐ordered spokes using continuous gradient ramps, but focus on eliminating acoustic noise instead of increasing sampling incoherence in time. Consequently, these ZTE trajectory designs limit the retrospective choices of temporal resolution to certain pre‐determined values.

Flexible temporal resolution in conventional sequences has proven clinically valuable in capturing dynamics of different time scales at optimal spatial resolutions within a single acquisition. Incoherent view orderings, such as golden‐angle [24] or bit‐reversed, provide the ability to resolve non‐periodic motion events, such as irregular breathing and bulk movement [25]. Such events would be averaged away by acquisitions that require periodic data binning into motion phases. In addition, these sampling patterns have enabled retrospective analysis of a single dataset at different time scales. For example, golden‐angle radial DCE can be analyzed at higher temporal resolutions to estimate the arterial input function and at lower temporal resolutions to estimate perfusion parameters [26]. However, these existing randomized view orderings cannot directly be applied to ZTE, due to the large angular distance between views and the challenge of unwanted gradient refocusing of residual coherences.

In this work, we propose Arc‐ZTE, a method in which the readout gradients are continuously slewed by a small amount during each TR, enabling large angular distances between successive spokes. We introduce a strategy to avoid gradient refocusing while maintaining temporally stable k‐space coverage, via an optimization framework to select sequential rotations of an arc‐shaped spoke. We then evaluate Arc‐ZTE in terms of k‐space coverage uniformity, sampling incoherence, and acoustic noise. Finally, we demonstrate its ability to achieve high spatiotemporal resolution in both static phantom and time‐resolved in vivo experiments.

## 2 Methods

### 2.1 Trajectory Design

Due to sequencer and gradient heating restrictions, ZTE sequences are typically segmented into groups of hundreds of TRs; between these segments, the gradients are ramped down. Segmented sequences also allow for interleaved contrast preparation modules [13]. Hence, in this section, we assume a segmented sequence and design continuous gradients for a single segment of 384 spokes. We rotate this designed segment in 3D by the golden angles [24] to produce a fully‐sampled trajectory. We assume that the prescribed resolution (or equivalently $k_{\max}$) and repetition time TR are determined by the operator.

#### 2.1.1 Arc Formulation

Each k‐space spoke is an arc of a planar circle with a radius r, which subtends an angle ϕ. We constrain 0°<ϕ<90° in this formulation. We choose k‐space spokes to be circular arcs because uniformly sampling along an arc corresponds to a constant magnitude gradient and slew rate. The first arc is taken to lie in the $k_{x}$–$k_{y}$ plane and can be described as:

(1) $$k_{x}\left(t\right)=\text{rsin}\left(\theta \left(t\right)\right)$$

(2) $$k_{y}\left(t\right)=\text{rcos}\left(\theta \left(t\right)\right)-r$$

(3) $$k_{z}\left(t\right)=0$$

where θ(t) is the angle subtended along the arc, ranging from θ(0)=0 to θ(TR)=ϕ. The corresponding gradients for this first arc are:

(4) $$G_{x}\left(t\right)=\frac{2\pi }{\gamma }\cdot \text{rcos}\left(\theta \left(t\right)\right)\cdot \frac{d\theta \left(t\right)}{\mathrm{dt}}$$

(5) $$G_{y}\left(t\right)=\frac{2\pi }{\gamma }\cdot -\text{rsin}\left(\theta \left(t\right)\right)\cdot \frac{d\theta \left(t\right)}{\mathrm{dt}}$$

(6) $$G_{z}\left(t\right)=0$$

where γ is the gyromagnetic ratio of hydrogen.

To design a single k‐space arc, we have two requirements to satisfy: (1) The arc must reach $k_{\max}$ at the end of the sampling time $t_{\mathrm{DAQ}}$, and (2) the arc must subtend ϕ at time TR. In general, $t_{\mathrm{DAQ}}\neq \mathrm{TR}$ due to system‐required dead‐time.

If we assume a constant gradient amplitude, then these two requirements translate to:

(7) $${\left(\text{rsin}\left(\theta \left(t\right)\right)\right)}^{2}+{\left(\text{rcos}\left(\theta \left(t\right)\right)-r\right)}^{2}=k_{\max}^{2}$$

(8) $$\theta \left(t\right)=\frac{t}{\mathrm{TR}}\cdot \phi$$

Hence, we can solve for r and the gradient amplitude |G|:

(9) $$r=k_{\max}\cdot \frac{1}{\sqrt{2\cdot \left(1-\cos\left(\phi \cdot \frac{t_{\mathrm{DAQ}}}{\mathrm{TR}}\right)\right)}}$$

(10) $$|G|=\frac{2\pi }{\gamma }\cdot r^{2}\cdot \frac{\phi }{\mathrm{TR}}$$

As expected, when the arc angle ϕ increases, a larger gradient amplitude is required to achieve the same resolution within the same TR (see Figure 6b).

#### 2.1.2 Trajectory Formulation

**FIGURE 2:**
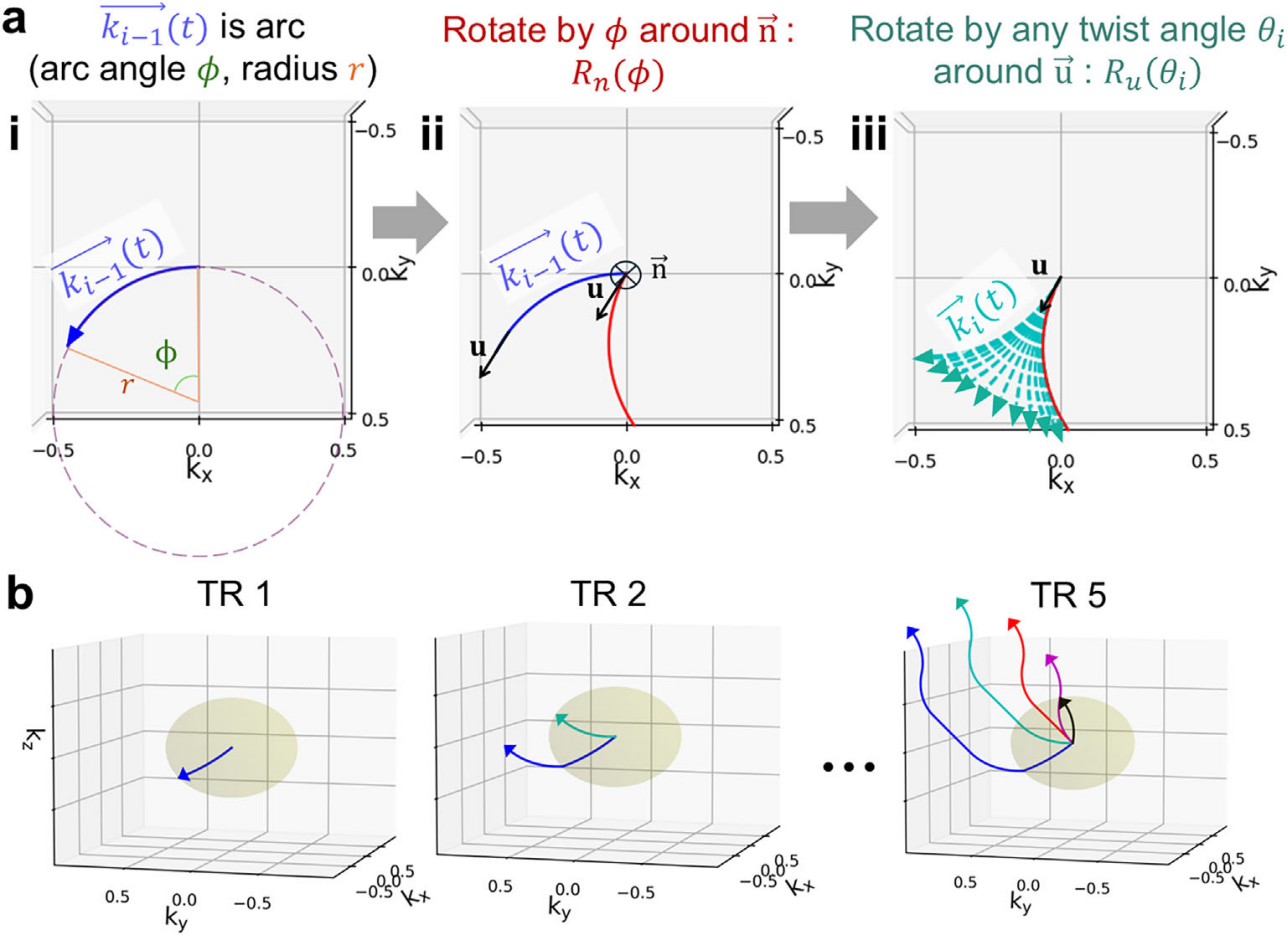
Trajectory parameterization and design. (a) (i) Our trajectory is parameterized as sequential rotations of an arc, so the i−1th spoke is plotted here (dark blue). The arc subtends angle ϕ of a circle with radius r. (ii) In order for gradients to be continuous between TRs, the start direction of the ith spoke must be equal to the end direction of the i−1th spoke; here this direction is labeled u. A first rotation $R_{n}\left(\phi \right)$ ensures that the ith spoke starts in direction u; here the red line illustrates the result of this first rotation. (iii) Another rotation $R_{u}\left(\theta_{i}\right)$ determines the end direction of the ith spoke. A rotation by any twist angle $\theta_{i}$ around u creates a valid spoke, so the choice of $\theta_{i}$ per TR is a degree‐of‐freedom. The different valid options for the ith spoke are plotted here as dotted turquoise lines. (b) Each selected spoke direction determines how previously excited coherences evolve and whether these previous coherences dephase outwards in k‐space, as shown here, or refocus towards the center of k‐space. Tracking these evolving coherences during the trajectory design ensures no undesired gradient refocusing occurs.

To maintain continuity of readout gradients through the segment, we formulate the trajectory as sequential rotations of the arc spoke, as follows:

(11) $$k_{i}\left(t\right)=R_{u}\left(\theta_{i}\right)\cdot R_{n}\left(\phi \right)\cdot k_{i-1}\left(t\right)$$

where $k_{i}\left(t\right)$ is the ith k‐space spoke, $R_{n}\left(\phi \right)$ is an in‐plane rotation by arc angle ϕ around the normal n, and $R_{u}\left(\theta_{i}\right)$ is a rotation by an arbitrary twist angle $\theta_{i}$ (see Figure 2a).

$R_{n}\left(\phi \right)$ is necessary to ensure continuity of the gradients between TRs. The ith spoke must start in the same direction that the i−1th spoke ends, and we name this direction u. Although the starting direction of the ith spoke is fixed, the ending direction is a degree‐of‐freedom. Any rotation by any angle $\theta_{i}$ around u would yield a viable option for the ith spoke that satisfies gradient and slew constraints. Therefore, the result forms a “cone” of possible options for the ith spoke, from which to select (see u in Figure 2a).

Rotation matrices are a convenient way to parameterize the k‐space trajectory because rotations in k‐space directly translate to rotations of gradient waveforms. If the rotation matrices for a given set of $\left\{\phi ,\theta_{i}\right\}$ are pre‐computed and stored on the scanner, they can be used for any scan prescription (i.e., FOV, resolution, and TR). Therefore, a method to compute the gradient waveforms for the entire Arc‐ZTE sequence could involve two steps: (1) computing the gradients for a single arc analytically based on the scan prescription, and (2) sequentially rotating the gradients of this arc using the pre‐computed rotation matrices.

For a segment of N spokes, our trajectory formulation has N+1 degrees of freedom: the arc angle ϕ and N per‐TR twist angles $\theta_{i}s$. The arc angle ϕ determines the curvature of each spoke, and hence, the slew rate amplitude of the sequence. Since it is known that slew rate directly affects acoustic noise, ϕ can be selected by the operator based on the patient's tolerance to acoustic noise.

**FIGURE 3:**
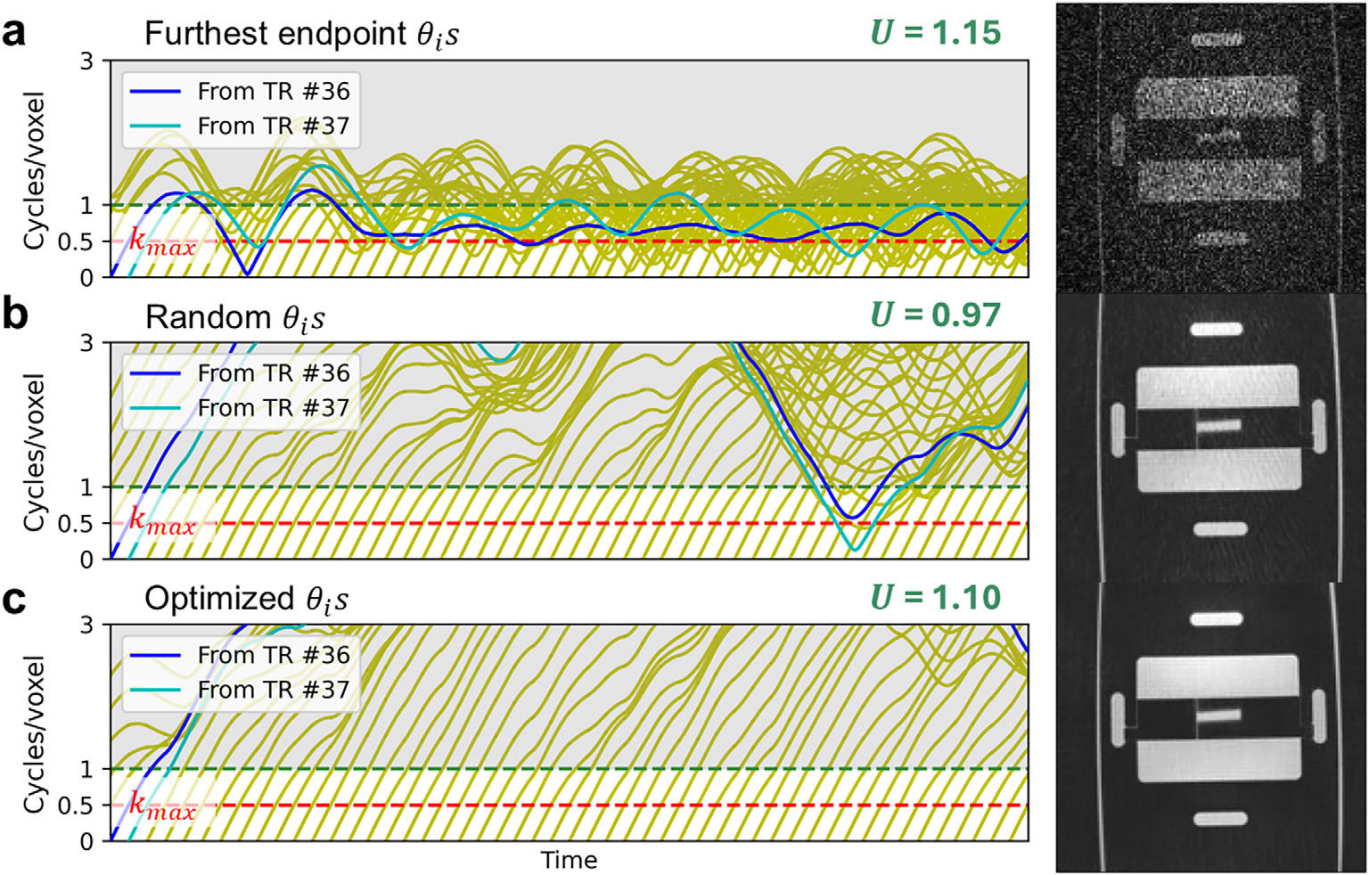
Selection of per‐TR twist angles $\theta_{i}s$. Coherence pathways of 50 TRs (starting with TR 36) for each scheme are displayed along with a slice from a gridding‐based reconstruction of a fully‐sampled phantom acquisition of each scheme. (a) Purely maximizing coverage uniformity over time, via selecting the $\theta_{i}$ that produces an endpoint furthest away from previous spokes, yields severe gradient refocusing and interference. (b) Randomly selecting $\theta_{i}s$ produces some instances of refocusing and compromises on coverage uniformity. Images contain some high‐frequency interference caused by the refocusing. (c) Our optimization‐based scheme allows us to select $\theta_{i}s$ that produce no instances of refocusing with greater coverage uniformity than random selection. Coverage uniformity U was calculated for 1 segment of 384 spokes of each scheme, and all schemes here use an arc angle ϕ=53°.

Given a fixed arc angle ϕ, we must select the per‐TR twist angles $\theta_{i}s$ carefully. For example, naively choosing $\theta_{i}s$ to maximize temporal k‐space coverage, by maximizing each spoke's distance from previous spokes, can result in severe artifacts due to gradient refocusing. This refocusing manifests as high‐frequency interference in the image (see Figure 3a). Alternatively, randomly choosing $\theta_{i}s$ will likely still generate instances of gradient refocusing, along with a reduction in k‐space coverage uniformity (see Figure 3b). Instead, we propose an optimization‐based approach as an informed way to select the $\theta_{i}s$, which will be described below.

#### 2.1.3 Optimization‐Based View Ordering

We present an optimization‐based approach to select the twist angles $\theta_{i}s$ per TR, which determines the view ordering within each segment. Our objective function simultaneously promotes k‐space coverage while penalizing gradient refocusing.

For each TR i, we minimize the following objective function:

(12) $$\max_{\theta_{i}}\;\sum_{j<i}∥k_{i}\left(t_{\mathrm{end}}\right)-k_{j}\left(t_{\mathrm{end}}\right){∥}_{2}+\lambda \sum_{t}\;\sum_{j<i}\log\left(∥k_{i}\left(t\right)+d_{j}{∥∥}_{2}\right)$$

where $k_{i}\left(t\right)$ is a function of $\theta_{i}$ via Equation (11), λ>0 is a tunable hyperparameter, and $d_{j}$ is the vector between the ith spoke and the jth previous coherence.

The first term in Equation 12 promotes k‐space coverage by maximizing the distance between the current spoke endpoint, $k_{i}\left(t_{\mathrm{end}}\right)$, to all previous spoke endpoints, $k_{j}\left(t_{\mathrm{end}}\right)$. The second term in Equation 12 penalizes gradient refocusing; it promotes current spokes that keeps previous coherences $k_{i}\left(t\right)+d_{j}$ far from the center of k‐space. We express the jth past coherence as $k_{i}\left(t\right)+d_{j}$ because all coherences experience the same gradients, and so, they evolve identically to the ith spoke, just at some distance $d_{j}$ away (see Figure 2b). The logarithm in Equation (12) penalizes coherences that refocus to < 1 cycle/voxel and heavily penalizes refocusing close to 0 cycle/voxel, or k‐space center.

Increasing the value of λ in Equation 12 decreases the relative importance of k‐space coverage uniformity over gradient refocusing. We selected λ via a grid search, with the chosen value being the smallest one that resulted in no instances of gradient refocusing within our segment length. The metric for gradient refocusing is further described in the next section.

Since this objective function Equation 12 is not computationally tractable, we discretized the $\theta_{i}$ space into increments of 1° and greedily selected the minimizer angle for each TR. Although the greedy discrete optimization is a heuristic without optimality guarantees, we empirically observed that it provides coverage uniformity close to that of naively choosing the furthest endpoints (see Figure 3). Additionally, we observed that our optimization‐based approach is effective across a range of arc angles 0°<ϕ<90°. In each tested case, our method produces no instances of gradient refocusing and approximately consistent k‐space coverage within our segment length.

#### 2.1.4 Quantifying Gradient Refocusing

Here, we define instances of gradient refocusing as instances where previously dephased coherences return to within < 1.25 cycle/voxel of phase. Although 1 cycle/voxel($=2k_{\max}$) corresponds to complete dephasing, we use a slightly larger threshold to avoid the accumulation of coherences exactly at 1 cycle/voxel. A time window of three TRs after excitation is allowed for each coherence to dephase before being included in this refocusing metric. Furthermore, we assume coherences are completely dephased by T2* after a time period of 200 ms, based on common T2* values in the body at both 1.5 and 3 T [27].

### 2.2 Sequence Design for DCE

DCE involves the injection of a T1‐shortening contrast agent [3], so an effective DCE sequence needs to provide sensitivity to the uptake of this agent. For DCE in the body, T1‐weighting and fat suppression are both required because fat has a short T1 [28], which may obscure enhancing tissue. We therefore include a preparation module to provide simultaneous T1‐weighting and fat suppression using a wideband saturation pulse, a delay time for T1 recovery, and a final fat‐selective inversion (see Figure S1 for pulse sequence diagram). The delay time after the saturation allows for recovery of short‐T1 tissues, especially contrast‐enhanced blood and fat, after which only fat is inverted. During the readout segment, fat recovers almost linearly, while contrast‐enhanced blood remains relatively constant. We selected the durations of the preparation and readout segments that yielded the average $M_{z}$ of fat to be approximately zero in each frame, hence enabling fat suppression. For 3 T systems, we chose 400 ms for preparation time and a 720 ms readout segment; for 1.5 T systems, we chose a 240 ms preparation time and a 540 ms readout segment. It should be noted that interleaving preparations at this rate reduced the scan efficiency of continuous ZTE by around 30%–35%.

### 2.3 Evaluation of Trajectory Characteristics

We evaluated Arc‐ZTE quantitatively on three metrics: coverage uniformity, sampling incoherence, and acoustic noise. For comparison, we considered the following common radial ZTE schemes: standard ZTE [15], radial ZTE with phyllotaxis interleaves (as formulated in [20]), and AZTEK [16]. Since AZTEK and phyllotaxis schemes both have a parameter space, we selected parameter sets that resulted in no gradient refocusing within 1 segment of 384 spokes, while still achieving pole‐to‐pole coverage. Figure S2 illustrates the trajectories and coherence pathways of the chosen schemes. For AZTEK, the parameters chosen were Shuffle = 1, Speed = 2, and Twist = 5. For the phyllotaxis scheme, we used a smoothness factor of 10 and 1 segment per interleave. Across all evaluations in this section, we fixed the acquisition parameters as 384 spokes per segment, 2.4 ms TR, 25.6 cm FOV, ±31.25 kHz BW, and 1 mm^3^ resolution.

We note that Figure S3 addresses comparisons where k‐space coverage uniformity across 384 spokes is kept comparable, and the resulting gradient refocusing is evaluated.

#### 2.3.1 K‐Space Coverage Uniformity

To analyze coverage uniformity of a set of ZTE k‐space spokes, we used the coverage uniformity metric U from Boucneau et al. [16] applied to the spoke endpoints. This metric is computed by generating a test point and computing its nearest distance to the spoke endpoints, then dividing by its nearest distance to a set of random points on the sphere. This computation is repeated and averaged over 10 000 test points. The resulting value is normalized to range from 0 to 2, where 2 indicates uniformly spaced points, 1 indicates randomly spaced points, and 0 indicates very clustered points. We computed this coverage uniformity metric across groups of successive spokes up to 8800 spokes (corresponding to a 21 s frame when TR = 2.4 ms) for Arc‐ZTE and comparison radial ZTE trajectories.

#### 2.3.2 Sampling Incoherence

We analyzed sampling incoherence by visualizing 2D and 1D cross sections of point spread functions (PSFs) for each comparison ZTE trajectory across groups of successive spokes. The maximum of the sidelobe‐to‐peak ratios (SPR) of these PSFs were also calculated via the following formula [29]: $\max_{i\neq j}|\frac{\mathrm{PSF}\left(i,j\right)}{\mathrm{PSF}\left(i,i\right)}|$. More incoherent sampling corresponds to lower SPR values, since large sidelobes are dispersed into noise‐like structures.

#### 2.3.3 Acoustic Noise

Acoustic energy was quantified using A‐weighted equivalent continuous sound pressure level [30] (LAeq). Our measurement setup consisted of an MR‐safe free‐field condenser microphone (Model 2541; Larson Davis Inc., Depew, NY) placed at the bore center at the location of the right ear. The microphone was connected via a 6 m cable to an integrating sound level meter (SLM 831C; Larson Davis Inc.) in the console room. All test pulse sequences were played out after switching off the transmit RF to avoid any unintended damage to the microphone and its preamplifier. We measured LAeq and A‐weighted acoustic spectra from 30 s sound samples and conducted all measurements within an hour to avoid any drifts.

We conducted the acoustic noise measurements for radial ZTE and Arc‐ZTE sequences using these parameters: 25.6 cm FOV, TR 2.4 ms, and 1 mm^3^ resolution. Gradient amplitude was 0.573 G/cm for the radial ZTE sequences and 0.574–0.612 G/cm for Arc‐ZTE with ϕ = 13°–83°. For comparison, we also measured the acoustic noise levels of 3D Cartesian SPGR and 3D radial UTE (bit‐reversed view ordering) sequences prescribed with the same FOV and resolution. Measurements were performed using a GE 3 T MR750w system.

### 2.4 Phantom and In Vivo Demonstrations

We qualitatively evaluated the spatiotemporal resolution enabled by Arc‐ZTE by acquiring and reconstructing static phantom and dynamic in vivo data. Acquisitions in these experiments used ±31.25 kHz BW, 2× readout oversampling, 3° flip angle, RF spoiling, and hard RF excitation.

Our reconstructions were required to handle challenges specific to ZTE acquisitions, namely missing samples at k‐space center and undesired signal from short‐T2 materials. In our acquisitions (using GE systems), each readout spoke missed the first 3 samples due to the dead time gap [31], which included time for half the RF pulse, T/R switch time, and coil ringdown time. We recovered this missing low‐frequency information implicitly by leveraging coil sensitivities in the reconstructions [32, 33]. We estimated the necessary coil sensitivities by applying ESPIRiT [34] on data from an initial WASPI [35] calibration scan. Furthermore, to avoid aliasing from short‐T2 materials located outside of the prescribed FOV, such as coil housing and cushions, we performed all reconstructions at a FOV 1.25×–2 larger than the prescribed FOV. For in vivo experiments, we additionally used ROVir [36] coil compression to suppress signal from outside the patient body.

#### 2.4.1 Phantom Experiment

To qualitatively characterize the flexibility of temporal resolution, we acquired phantom data for each ZTE scheme and reconstructed a single frame with different numbers of consecutive spokes. Data were acquired from an ACR phantom on a GE MR750w 3 T system with a GE 8‐channel HR brain array (model 2317112). Scan parameters were as follows: 25.6 cm FOV, 384 spokes/segment, 2.4 ms TR, 1 mm^3^ resolution. The fully‐sampled acquisition had 66 048 readout spokes.

Reconstructions were performed using an l1‐wavelet regularized iterative SENSE reconstruction, which was solved with polynomial‐preconditioned FISTA [37] and implemented with the BART framework [38]. Matrix size was 128 × 128 × 128 and reconstructions were conducted at 1.25× prescribed FOV. For this experiment, all phantom reconstructions used the same coil sensitivity maps.

#### 2.4.2 Time‐Resolved Lung Imaging Experiments

With IRB approval and informed consent, we acquired free‐breathing lung data from three patients, with each patient scanned using both Arc‐ZTE with ϕ = 53° and radial ZTE with phyllotaxis interleaves. To validate the resolved dynamics, we reconstructed low‐resolution image self‐navigators at spatial resolution (7.5 mm^3^) and temporal resolution of 200 spokes/frame (0.5 s/frame). We derived a motion estimate for each reconstructed frame via cross correlation of a 1D cross‐section through the liver‐diaphragm interface, using the first frame as the reference. To qualitatively check these motion estimates, they were compared to the bellows signal.

Furthermore, we performed higher resolution reconstructions with this patient data via an explicit low‐rank formulation, similar to the approach by Feng et al. [39]. The aforementioned low‐spatial‐resolution reconstructions were used to derive a temporal basis using an SVD. Using this temporal basis, we solved for the corresponding spatial coefficient images using a locally low‐rank regularized reconstruction. We empirically selected a rank of 8 for these reconstructions. Both low‐resolution and final reconstructions were solved with polynomial‐preconditioned FISTA [37] and ROVir coil compression [36] as a pre‐processing step.

These patient acquisitions were performed on a GE SIGNA‐Artist 1.5 T system with GE AIR coils placed anteriorly and posteriorly (41 channels total). Acquisition parameters were as follows: 30 cm^3^ FOV for Cases 1 and 2, 34 cm^3^ FOV for Case 3, 1.1 mm^3^ resolution for Cases 1 and 2, 1.2 mm^3^ resolution for Case 3. Reconstructions were performed at 2× prescribed FOV; matrix size was 80 × 80 × 80 for the low‐resolution reconstructions and 256 × 256 × 256 for the higher resolution reconstructions.

#### 2.4.3 DCE Experiment

To evaluate if Arc‐ZTE could also resolve rapid contrast dynamics, we conducted a DCE acquisition for 188 s using Arc‐ZTE with ϕ = 53°. A dose of 0.05 mmol/kg of gadopiclenol was administered to a pediatric patient at the 30 s mark. Acquisitions were performed on a GE SIGNA‐Premier 3 T system with GE AIR coils placed anteriorly and posteriorly (41 channels total). Acquisition parameters were as follows: 24 cm^3^ FOV, 0.8 mm^3^ resolution, 2.8 ms TR, 256 spokes/segment, and 400 ms prep time.

We reconstructed the data with an explicit low‐rank formulation [39] in the same way as described in Section 2.4.2, but at a temporal resolution of 256 spokes/frame (1.1 s/frame). We empirically selected a rank of 16 for the temporal basis derived from an SVD of the low‐resolution reconstructions (5 mm^3^). Reconstructions were performed at 1.75× prescribed FOV. All other reconstruction details were the same as described in Section 2.4.2.

## 3 Results

### 3.1 Results From Evaluation of Trajectory Characteristics

**FIGURE 4:**
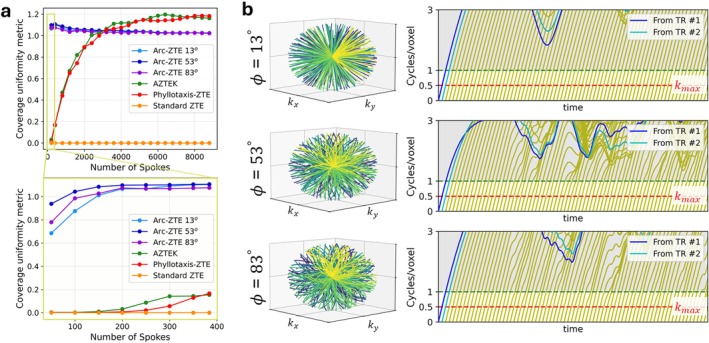
Evaluation of coverage uniformity, sampling trajectory, and coherence evolution for different arc angles. (a) Coverage uniformity U across different number of spoke endpoints was calculated for Arc‐ZTE with different arc curvatures (13°, 53°, and 83°) and for comparison radial ZTE schemes. Arc‐ZTE schemes had stable coverage uniformity across all tested spokes, with 53° having the most stable coverage, even at < 200 spokes. (b) Trajectory for one Arc‐ZTE segment of 384 spokes (arc angles 13°, 53°, and 83°) is plotted, where color of each spoke represents the time of acquisition. The corresponding coherence pathways for 70 successive TRs of these schemes are also plotted. The optimization‐based trajectory design effectively controls the evolving coherences to remain dephased (gray region of plot), even for large arc curvatures like 83°. Figure S1 illustrates the coherence pathways for the comparison radial ZTE schemes.

Figure 4a demonstrates that Arc‐ZTE improved the temporal stability of k‐space coverage uniformity over comparison radial ZTE schemes. For Arc‐ZTE, the coverage uniformity U was approximately constant across the tested numbers of consecutive spokes, while the AZTEK and phyllotaxis schemes only matched this coverage at around 3000 consecutive spokes (corresponding to a frame of 7.2 s if TR = 2.4 ms). As expected, standard ZTE did not achieve comparable coverage uniformity in any of the tested durations. Between the three tested arc angles, Arc‐ZTE with 53° demonstrated the highest coverage uniformity, especially at < 200 spokes.

**FIGURE 5:**
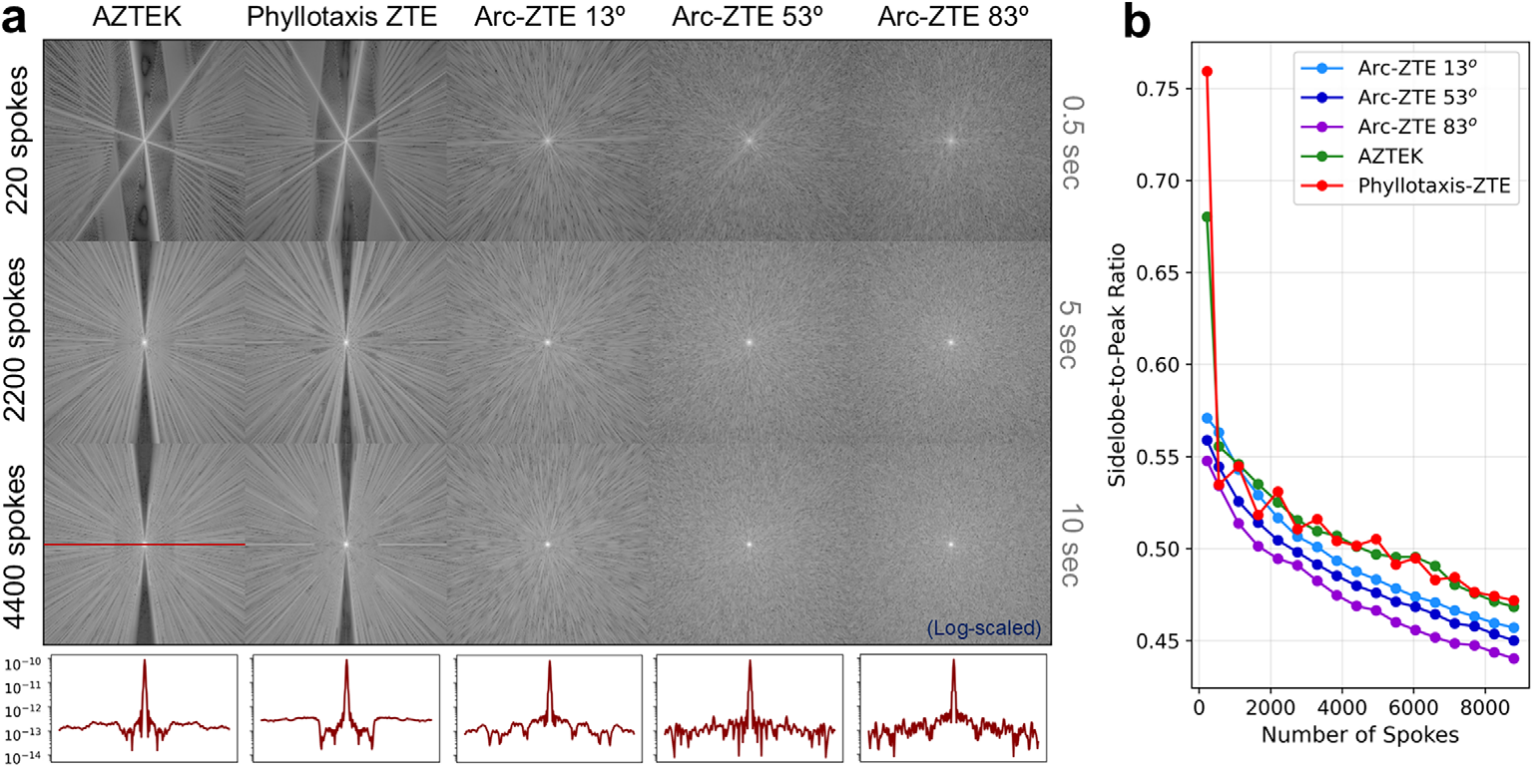
Evaluation of sampling incoherence. (a) The 2D cross sections ($k_{y}$–$k_{z}$) of the radial ZTE and Arc‐ZTE point spread functions (PSFs) are plotted for frames of different number of spokes. Radial ZTE PSFs show streaking and coherent structures, while the Arc‐ZTE PSF is noise‐like in all directions, especially as the arc angle increases. An example 1D cross section of each PSF is plotted in red, demonstrating the noise‐like sidelobes for Arc‐ZTE. (b) To quantify incoherence, the sidelobe‐to‐peak ratio (SPR) for each 3D PSF is plotted across frames of different number of spokes. Arc‐ZTE achieves smaller SPR values than radial ZTE schemes, with smaller SPR values for increased arc curvature.

The improved sampling incoherence of Arc‐ZTE is illustrated with the point spread functions (PSF) in Figure 5a. Arc‐ZTE demonstrated a noise‐like PSF in all directions, while all the radial ZTE schemes generated PSFs with visible streaks and coherent structures, especially at a lower number of spokes. Increasing the arc angle improved the noise‐like quality of the Arc‐ZTE PSF, as seen more clearly in the sidelobes of the example 1D cross sections of the PSFs. In 5b, the sidelobe‐to‐peak ratios (computed from the 3D PSFs) were consistently smaller for Arc‐ZTE than radial ZTE across all tested numbers of spokes. Increasing the arc curvature decreased the sidelobe‐to‐peak ratio, hence increasing the sampling incoherence.

**FIGURE 6:**
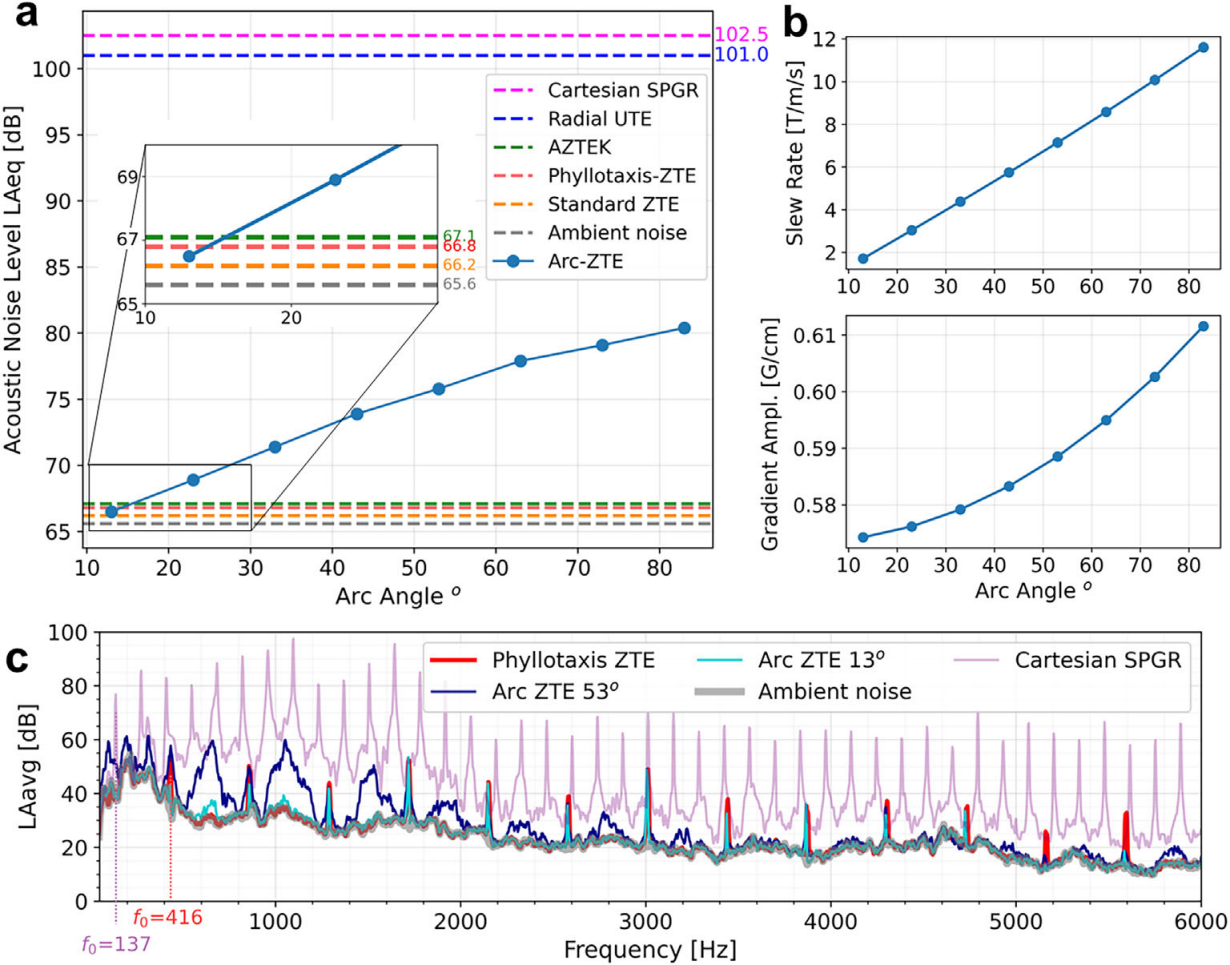
Evaluation of acoustic noise. (a) Acoustic noise measurements (LAeq) acquired on an integrating sound level meter with a microphone placed at isocenter at the position of the right ear. All sequences used 25.6 cm FOV and 1 mm^3^ resolution. For Arc‐ZTE, LAeq was within the range of 0.9–14.8 dB above ambient noise, with a consistent increase as the arc curvature increased. Ambient noise LAeq was 65.6 dB. (b) Slew rate and gradient amplitude across arc angles of the Arc‐ZTE sequences used in measurements. (c) Acoustic spectra generated by the measured sequences and ambient noise. Both the ZTE and Cartesian SPGR sequences generated harmonics with a fundamental frequency of 1/TR (here 416 Hz for the 2.4 ms ZTE TR and 137 Hz for the 7.3 ms SPGR TR), while Arc‐ZTE generated additional low frequency components below 2 kHz.

Figure 6a shows that Arc‐ZTE increased the sound pressure level over the comparison radial ZTE schemes, but was significantly quieter than conventional sequences. Comparison radial ZTE schemes resulted in LAeq of 66.2–67.1 dB, Arc‐ZTE resulted in 66.5–80.4 dB over arc angles 13°–83°, while conventional 3D sequences were measured at 101.0–102.5 dB. Ambient noise level was 65.6 dB.

Figure 6c illustrates that the acoustic spectra of both ZTE and Cartesian SPGR are dominated by harmonics with fundamental frequencies of 1/TR (416 Hz for the 2.4 ms ZTE TR, and 137 Hz for the 7.3 ms Cartesian SPGR TR). In addition, Arc‐ZTE contained low‐frequency acoustic resonance bands, which increased in amplitude as the arc curvature increased.

### 3.2 Results From Phantom and In Vivo Demonstrations

**FIGURE 7:**
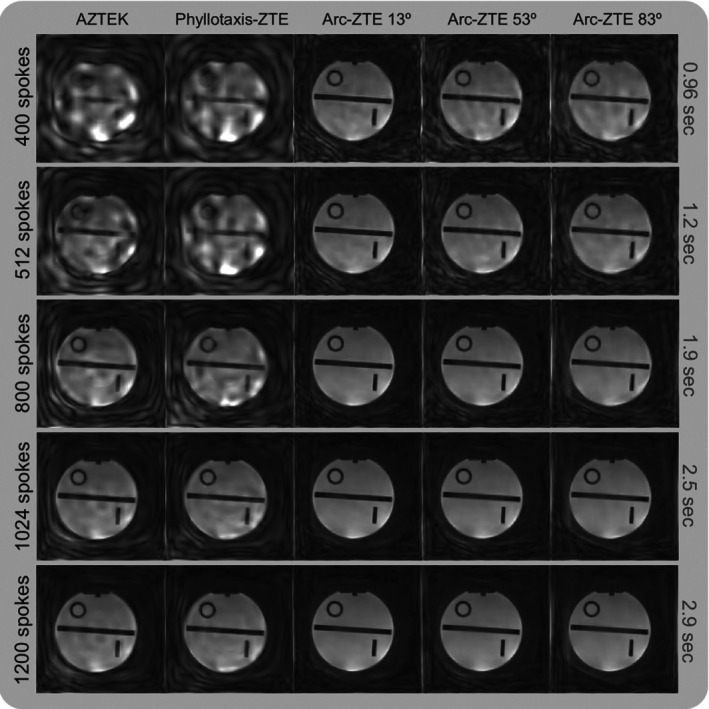
Phantom reconstructions using different numbers of consecutive spokes. AZTEK and phyllotaxis schemes have image quality that significantly degrades at 400 spokes. Arc‐ZTE schemes have superior image quality in all tested cases, where the difference is most obvious at a smaller number of spokes. Although differences between the Arc‐ZTE angles are minor, there is some additional blurring observed with 83° at 400 and 512 spokes.

The phantom reconstructions across different numbers of spokes are shown in Figure 7. At all tested frame durations, the image quality from Arc‐ZTE schemes was visually much sharper than that from the radial ZTE schemes. For radial ZTE, the image quality degraded significantly between 400 and 1200 spokes/frame. Between the three tested arc angles, there was no significant difference in image quality, although reconstructions from the 83° arc angle had slightly more blurring at 400 and 512 spoke durations.

**FIGURE 8:**
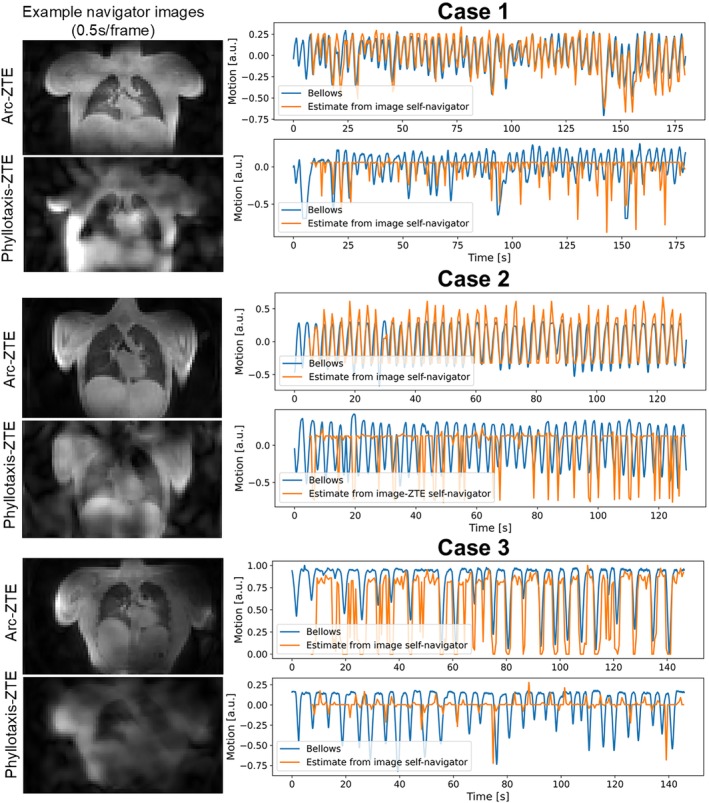
Low‐resolution image self‐navigators across three patient cases. For each, an example frame from the low‐resolution reconstruction and the motion estimates are displayed. There is a stark difference in image quality between Arc‐ZTE and the phyllotaxis ZTE scheme for each of the cases. Motion estimates derived from the Arc‐ZTE reconstructions match the bellows signal well, demonstrating that expected breathing dynamics are resolved. Motion estimates from the phyllotaxis reconstructions appear as mostly zero with noise spikes, since the navigator images fail to capture the expected motion due to the extreme level of artifacts. Dynamics can be visualized in Video S1.

**FIGURE 9:**
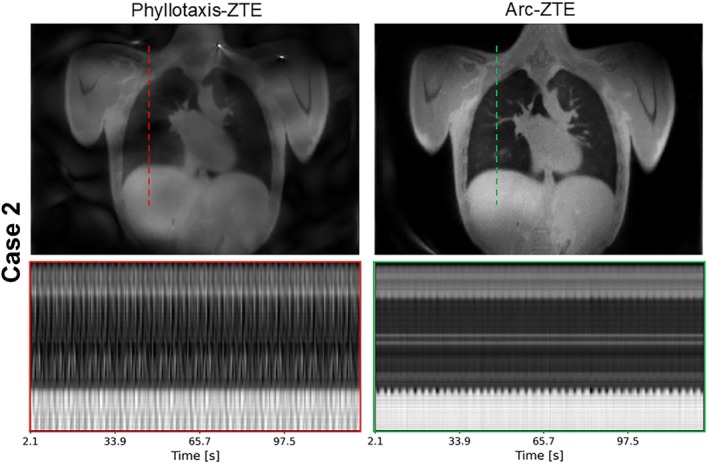
Time‐resolved reconstruction of patient case 2 at 0.5 s/frame. Reconstruction used an explicit low‐rank formulation, where the temporal basis components were computed from low‐resolution reconstructions. We empirically selected a rank of 8. Arc‐ZTE reconstruction demonstrates the expected breathing dynamics with superior image quality compared to the phyllotaxis scheme, where the images are significantly more blurry. Dynamics can be further visualized in Video S2. Artifacts near the edges of this extended FOV can be seen in certain slices from both reconstructions and are attributed to gradient roll‐off, as shown in Figure S5.

For the free‐breathing dataset, Figure 8 demonstrates that the low‐resolution Arc‐ZTE reconstructions (at 0.5 s/frame or 200 spokes/frame) were able to capture breathing dynamics that matched well with the respiratory bellows. This was visible in both normal breathing patterns (Case 1 and 2) and abnormal patterns (Case 3). In contrast, radial ZTE with phyllotaxis interleaves at this temporal resolution resulted in poor image quality and was unable to capture the expected breathing dynamics. For Case 2, the time‐resolved reconstructions at full spatial resolution are shown in Figure 9. The phyllotaxis scheme produced blurry images that did not capture the expected breathing dynamics, while Arc‐ZTE resulted in much higher image quality and physiologically consistent dynamics. Dynamics can be visualized in Videos S1 and S2.

**FIGURE 10:**
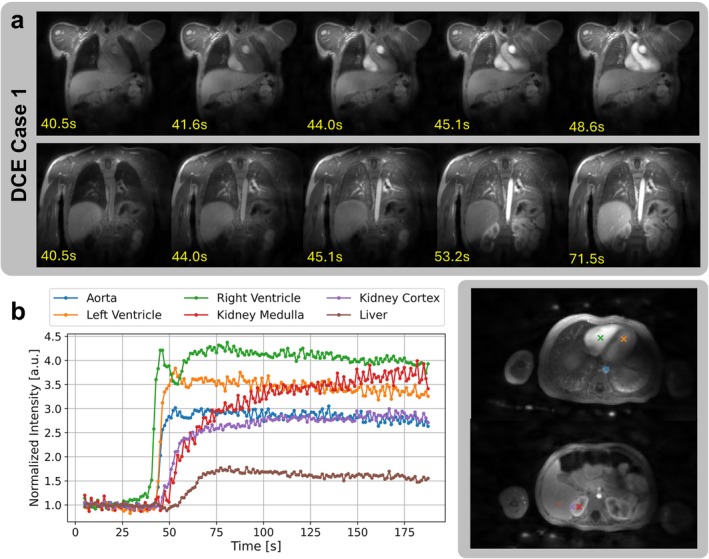
Results from the DCE experiment using T1‐prepared Arc‐ZTE. Contrast agent was administered 30 s after the scan was started and data was acquired for 188 s. (a) Two sets of coronal slices from the reconstruction captured the rapid dynamics of contrast arrival and demonstrated the expected delay between right and left ventricle and between kidney cortex and medulla. Dynamics can be visualized in Video S3. (b) Enhancement curves from voxels of different regions of interest at indicated locations (right images). Curves were physiologically consistent, and low‐rank constraint acted as a filter for respiratory motion. Figure S6 evaluates the resolved respiratory motion in this reconstruction.

Figure 10 shows example frames from the reconstruction of the DCE acquisition with Arc‐ZTE. These reconstructions demonstrated that Arc‐ZTE was able to resolve rapid, physiologically consistent dynamics, such as the rapid wash‐in of the ventricular cavities. Enhancement curves showed the expected delay between right and left ventricles, and between kidney cortex and medulla. Dynamics can be visualized in Video S3, where some minor fluctuations are visible that typically occur in low‐rank reconstructions [25].

## 4 Discussion

In this paper, we proposed Arc‐ZTE, a method that enables flexible, dynamic, near‐silent MRI by improving temporal sampling of ZTE without inducing gradient refocusing. Our curved readouts allow successive spokes to cover larger angular distances without using large slew rates. Consequently, sampling incoherence and temporal k‐space coverage uniformity improve over radial ZTE methods, leading to improvements in achievable temporal resolutions over existing radial ZTE methods.

Although we formulate the curved readouts as arcs of a planar circle, this is merely a design choice and not necessary to use our framework in Section 2.1.3. This framework, which tracks the history of previous coherences to design the sampling trajectory, could still be used for other types of curved readouts, such as 3D space curves or hybrid radial‐arcs. Moreover, even with radial spokes, explicitly tracking the evolving coherences can help avoid undesired instances of gradient refocusing (see Figure S2). Therefore, one of the main contributions of our work is a trajectory design that explicitly accounts for spoiling persistent coherences in ZTE.

Our optimization‐based approach to select per‐TR twist angles $\theta_{i}s$ empirically worked sufficiently well for our segment length of 384 spokes; however, this segment length can be increased for different system sequencers. To design longer segments, the greedy algorithm to select $\theta_{i}s$ would be constrained by longer histories of coherences. With these longer designs, it could be beneficial to relax the second term in Function 12 by weighting the previous coherences by their amount of experienced T2* decay. We used a discretized greedy algorithm for its simplicity, but it is possible that a more optimal solution could be calculated with a stochastic greedy approach or by jointly optimizing over the $\theta_{i}$'s for all TRs. These alternate algorithms would allow for better exploration of the solution space, at the expense of algorithm simplicity.

Figure 6a shows that our design of Arc‐ZTE yields a clear relationship between sound pressure level (LAeq) and arc curvature ϕ. Hence, an operator could easily lower the acoustic level of the sequence for more sound‐sensitive patients with a tradeoff in sampling incoherence. Further investigation could potentially lower the sound pressure levels by modifying Arc‐ZTE gradient waveforms to avoid mechanical resonances of the system. In addition to measuring overall sound energy, Figure 6c shows that the acoustic noise quality changes between radial and curved spokes. Since LAeq provides a measure of sound energy, this metric might not directly correspond to how unpleasant the acoustic noise is perceived to be. With Arc‐ZTE, we empirically observed that the acoustic noise quality included a distinct low pitch rumble since the gradients are slewing continuously, making the noise sound broadband. In contrast, the phyllotaxis and AZTEK sequences had a strong tonal sound, where the perceived frequency corresponded to 1/TR (= 440 Hz for a TR of 2.3 ms). Psychoacoustic studies have shown that humans are generally more sensitive to tonal noise than broadband noise [40], indicating that the effect of the different ZTE sound qualities on patient comfort could be further investigated.

Our analysis demonstrates that Arc‐ZTE can be an overarching strategy for continuous, near‐silent acquisition in dynamic imaging. Unlike radial ZTE schemes [16, 20] where parameters are selected based on pre‐defined binning strategies, Arc‐ZTE trajectory design does not depend on the dynamics to be resolved. Arc‐ZTE enables retrospective binning at a range of temporal resolutions due to its temporal stability of k‐space coverage uniformity, as shown in Figure 4a. In addition, Arc‐ZTE provides improved sampling incoherence, as shown in Figure 5, making it well‐suited for sparsity‐based and low‐rank priors. Due to the combination of incoherent efficient sampling and low‐rank priors, our in vivo demonstrations achieved high spatiotemporal resolutions, as illustrated in Figures 8, 9, 10.

Incoherent sampling from Arc‐ZTE could also prove beneficial for static applications. Figure 7 demonstrates that with high undersampling ratios, the image quality from Arc‐ZTE can improve over existing radial ZTE schemes. The phantom experiment leveraged the incoherent sampling of Arc‐ZTE by reconstructing using a sparsity‐based prior. Moreover, when magnetization preparation is interleaved between ZTE segments, the well‐distributed sampling of Arc‐ZTE helps average out recovery during the readout segment [41]. We have leveraged this fact in our DCE demonstration, illustrated in Figure 10, which interleaves a T1‐preparation module between Arc‐ZTE segments of duration 0.7 s. Other applications could use Arc‐ZTE to improve the fidelity of near‐silent parameter mapping [42]. However, for acquisitions with more than 3000 spokes, Arc‐ZTE achieves a lower coverage uniformity than radial ZTE schemes (see Figure 4a), likely due to our golden‐angle rotations of the designed segment. While this was a simple choice to promote incoherent, more optimized rotations would likely improve global coverage uniformity.

Using curved spokes instead of radial spokes introduces some additional considerations for application to existing ZTE techniques. Several works [32, 43] correct the excitation profile across the radial ZTE spokes by leveraging 1D sub‐problems along each spoke dimension. While we did not correct the excitation profile as short hard RF pulses were used, future work with longer RF pulses would need to perform this correction in 3D. Furthermore, gradient delay correction is critical with curved spokes, as delays between the gradients and the RF pulse affect the arc spoke direction. In contrast, minor timing errors between the constant gradients of radial ZTE and the RF pulse do not affect the radial spoke direction. In this work, we tuned timings empirically by examining the image quality resulting from trajectories of a set of different delays; an example of tuning gradient delays is provided in Figure S7. However, more thorough modeling of the system response to Arc‐ZTE gradients would be beneficial, especially to account for gradient heating [44] across long duration acquisitions.

## 5 Conclusion

This work introduces Arc‐ZTE, a method to improve temporal k‐space sampling for near‐silent Zero‐TE imaging via readout gradients that are continuously slewed by a constant, small slew rate. Our trajectory design directly promotes k‐space coverage over time while penalizing gradient refocusing. We demonstrated quantitative improvements in sampling incoherence and temporal k‐space coverage uniformity over existing radial ZTE schemes. Arc‐ZTE was substantially quieter than conventional dynamic MRI sequences by 20–35 dB; however, increasing the arc curvature raised acoustic noise levels beyond those of the comparison radial ZTE schemes by up to 14 dB. Phantom and in vivo demonstrations illustrated the ability of Arc‐ZTE to achieve high spatiotemporal resolutions. Overall, this work could significantly improve the clinical success of dynamic imaging among neonatal, pediatric, and sound‐sensitive patients.

## Funding

This work was supported by National Institutes of Health Grant/Award Numbers: R01EB036127, R01EB009690, R01HL173035; GE Healthcare.

## Conflicts of Interest

Michael Lustig receives research support from GE Healthcare.

## Supporting information


**Figure S1:** T1‐prepared Arc‐ZTE with fat suppression used for the DCE experiment at 3 T. Between every readout segment of 256 continuous spokes, the readout gradients are ramped down and a custom preparation module is played out. The preparation consists of a wideband saturation to crush all tissues, followed by a 400 ms time period $T_{\text{prep}}$, where fat and enhanced blood recover by a large amount. Finally, a fat‐selective inversion is applied, such that fat recovers almost linearly through the readout segment and its signal averages out close to zero.
**Figure S2:** Trajectories of 1 segment and magnetic coherence pathways of 85 TRs across different parameter sets for the AZTEK and phyllotaxis schemes. Trajectories are plotted for 1 segment of 384 spokes, where color indicates time of acquisition. Refocusing is visible for phyllotaxis scheme with smoothness = 9 and for AZTEK with Shuffle = 1, Speed = 3, and Twist = 5. Refocusing occurs even though the radial spokes are arranged in a smooth path around k‐space.
**Figure S3:** Trajectories and coverage uniformity U of 1 segment of 384 spokes, along with magnetic coherence pathways of 100 TRs, across interleaved view‐ordering schemes. Color of the trajectory spokes indicate time of acquisition. Although tiny golden angle schemes can achieve comparable coverage uniformity to Arc‐ZTE within 384 spokes, they result in several instances of gradient refocusing. Similarly, phyllotaxis schemes with high slew rates than that used in the main manuscript can also achieve comparable coverage uniformity but also result in instances of gradient refocusing.
**Figure S4:** Comparison of a 2D cross‐section from the 3D point spread functions of fully‐sampled ZTE trajectories. Here, the full trajectory consists of 66 048 spokes, which is the empirical Nyquist rate for a matrix size of 256, and corresponded to a scan time of around 2 min and 47 s. The Arc‐ZTE PSFs continue to appear noise‐like, due to the sampling incoherence throughout the complete trajectory.
**Figure S5:** Example slices demonstrating bright artifacts near the edges of the extended FOV. Slices were taken from time‐resolved reconstruction from Case 2 for both ZTE schemes. These artifacts are hypothesized to arise from the gradient roll‐off, which collapses out‐of‐FOV signal into bright lines. In certain slices, this signal collapse appears as bright points, which appear larger in the phyllotaxis reconstruction due to the lower image quality. This signal can be attributed to both the surrounding tissue and plastic, due to the non‐selective excitation and the zero echo time.
**Figure S6:** Demonstration of respiratory motion in DCE experiment reconstructed at 1.1 s/frame. (a) Plot of the 4 most significant temporal basis components, which were extracted from an SVD of the low‐resolution reconstruction. Components 1, 2, and 3 seem to capture the baseline and general contrast enhancement dynamics, while Component 4 appears to capture the respiratory motion. (b) Time‐curves of two ROIs from the reconstruction: one from the diaphragm region and one from the right ventricle. The explicit low‐rank constraint resolves the respiratory motion in regions where it is significant, such as the diaphragm, and filters out the motion elsewhere, such as the left ventricle.
**Figure S7:** Empirical procedure to tune the timing offsets between the DAQ and gradients, by interpolating sampling coordinates along a fixed trajectory. A negative timing offset here indicates the gradient waveforms are delayed with respect to the DAQ (data acquisition), while a positive offset indicates the converse. Based on these images, a −2μs offset of the gradients was used for this system. It should be noted that tuning the timing offset between the RF and gradients would instead involve generating new trajectory coordinates.

**Video S1:** Low‐resolution image self‐navigator reconstructions of free‐breathing lung acquisitions at 0.5 s/frame temporal resolution and 7.5 mm^3^ spatial resolution from three patient cases. Although there is some flickering in the Arc‐ZTE reconstructions, the respiratory motion can be visualized well. In contrast, the reconstructions of the Phyllotaxis‐ZTE data contain a significant level of artifacts, making it difficult to observe the respiratory motion.

**Video S2:** Reconstruction of free‐breathing lung acquisitions from patient Case 2 reconstructed at 0.5 s/frame temporal resolution with a rank 8 temporal subspace. The reconstruction of the Arc‐ZTE data resolves respiratory motion, although the low‐rank constraint does not completely capture the motion of the vessels. Finer pulmonary vessels are also visible. However, the same reconstruction with Phyllotaxis‐ZTE data is unable to mitigate undersampling artifacts or resolve respiratory motion.

**Video S3:** Reconstruction of the near‐silent DCE experiment acquired with Arc‐ZTE and reconstructed at 1.1 s/frame temporal resolution with a rank 16 temporal subspace. (a) Rapid contrast uptake is visualized in the expected order of right ventricles, lungs, left ventricle, then descending aorta. (b) Rapid contrast enhancement in the lungs and aorta is visualized, followed by slower enhancement in the kidney cortex then medulla. Minor unexpected fluctuations are observed in the videos, likely due to the low rank constraint.

## Data Availability

Code to reproduce results in this work is openly available on Github at https://github.com/mikgroup/arc_zte.git.
