# Supplementary material for: Arc‐ZTE: Continuously‐Slewed Zero‐TE Imaging With Incoherent Temporal Sampling for Near‐Silent Dynamic MRI
Source: Magn Reson Med. 2026 Jun 16;96(4):1726–40. doi: 10.1002/mrm.70477 (PMC13418941; doi:10.1002/mrm.70477)
Supplement: Supplementary file 1 — Figure S1: T1‐prepared Arc‐ZTE with fat suppression used for the DCE experiment at 3 T. Between every readout segment of 256 continuous spokes, the readout gradients are ramped down and a custom preparation module is played out. The preparation consists of a wideband saturation to crush all tissues, followed by a 400 ms time period Tprep, where fat and enhanced blood recover by a large amount. Finally, a fat‐selective inversion is applied, such that fat recovers almost linearly through the readout segment and its signal averages out close to zero. Figure S2: Trajectories of 1 segment and magnetic coherence pathways of 85 TRs across different parameter sets for the AZTEK and phyllotaxis schemes. Trajectories are plotted for 1 segment of 384 spokes, where color indicates time of acquisition. Refocusing is visible for phyllotaxis scheme with smoothness = 9 and for AZTEK with Shuffle = 1, Speed = 3, and Twist = 5. Refocusing occurs even though the radial spokes are arranged in a smooth path around k‐space. Figure S3: Trajectories and coverage uniformity U of 1 segment of 384 spokes, along with magnetic coherence pathways of 100 TRs, across interleaved view‐ordering schemes. Color of the trajectory spokes indicate time of acquisition. Although tiny golden angle schemes can achieve comparable coverage uniformity to Arc‐ZTE within 384 spokes, they result in several instances of gradient refocusing. Similarly, phyllotaxis schemes with high slew rates than that used in the main manuscript can also achieve comparable coverage uniformity but also result in instances of gradient refocusing. Figure S4: Comparison of a 2D cross‐section from the 3D point spread functions of fully‐sampled ZTE trajectories. Here, the full trajectory consists of 66 048 spokes, which is the empirical Nyquist rate for a matrix size of 256, and corresponded to a scan time of around 2 min and 47 s. The Arc‐ZTE PSFs continue to appear noise‐like, due to the sampling incoherence throughout the complete tra [file MRM-96-1726-s002.pdf]

Supplementary Figures

1 T1 preparation for DCE experiment

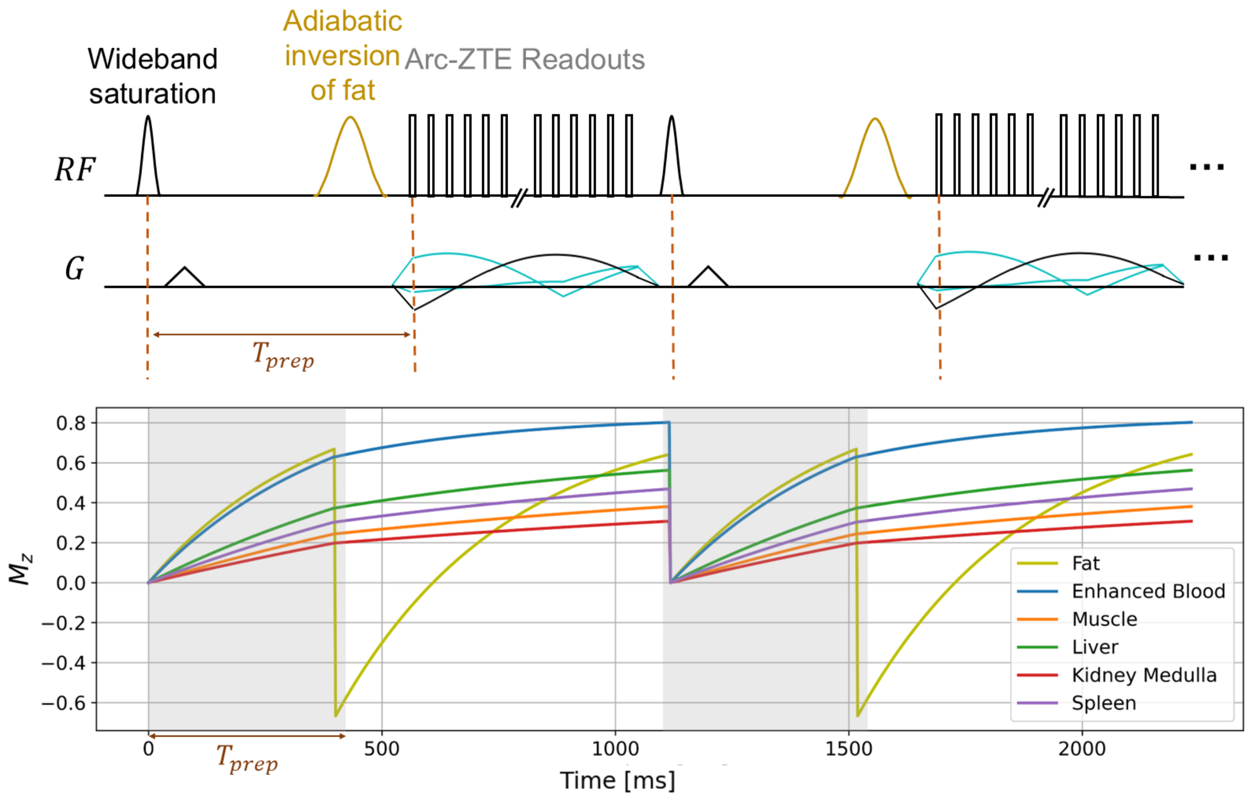

Figure S1: T1-prepared Arc-ZTE with fat suppression used for the DCE experiment at 3T. Between every readout segment of 256 continuous spokes, the readout gradients are ramped down and a custom preparation module is played out. The preparation consists of a wideband saturation to crush all tissues, followed by a 400ms time period T_{prep}, where fat and enhanced blood recover by a large amount. Finally, a fat-selective inversion is applied, such that fat recovers almost linearly through the readout segment and its signal averages out close to zero.

2 Parameter selection for comparison radial ZTE schemes

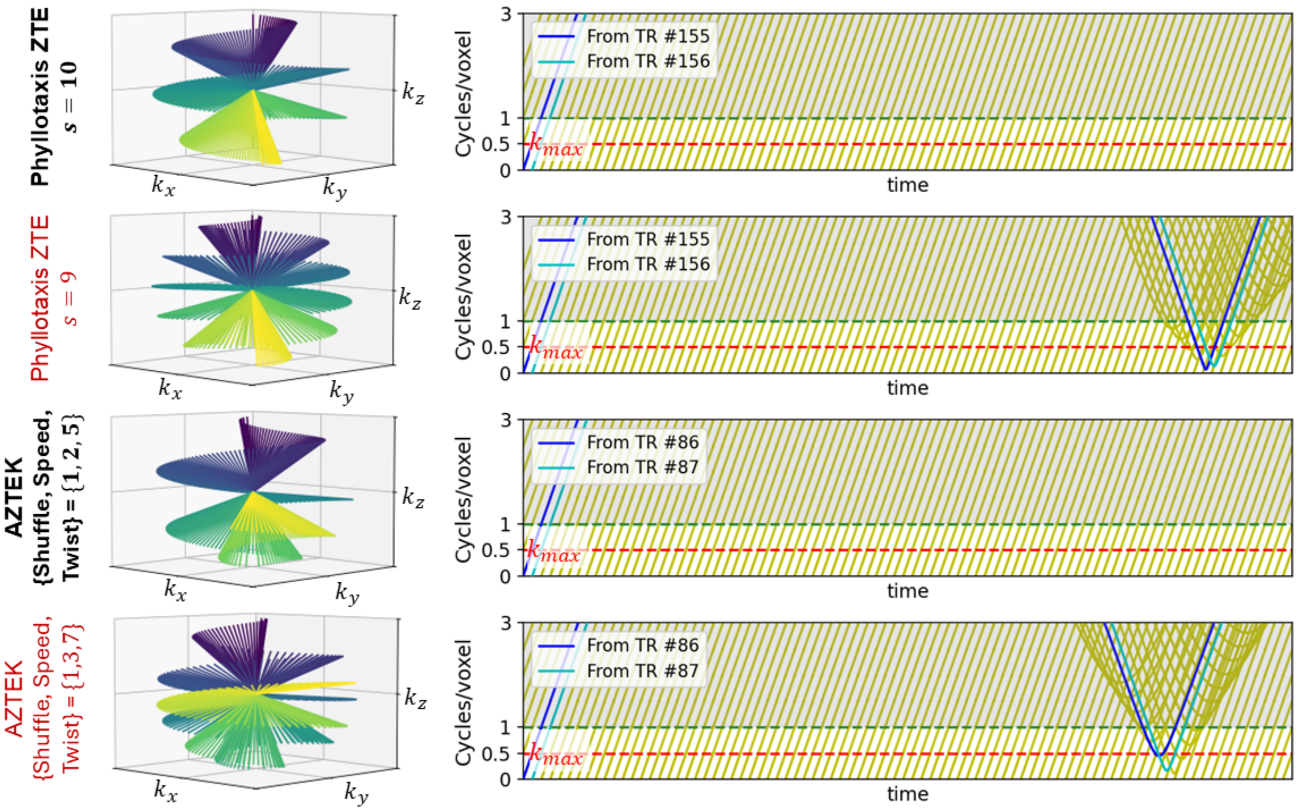

Figure S2: Trajectories of 1 segment and magnetic coherence pathways of 85 TRs across different parameter sets for the AZTEK and phyllotaxis schemes. Trajectories are plotted for 1 segment of 384 spokes, where color indicates time of acquisition. Refocusing is visible for phyllotaxis scheme with smoothness=9 and for AZTEK with Shuffle=1, Speed=3, and Twist=5. Refocusing occurs even though the radial spokes are arranged in a smooth path around k-space.

3 Refocusing and coverage comparison with tiny golden angle view ordering

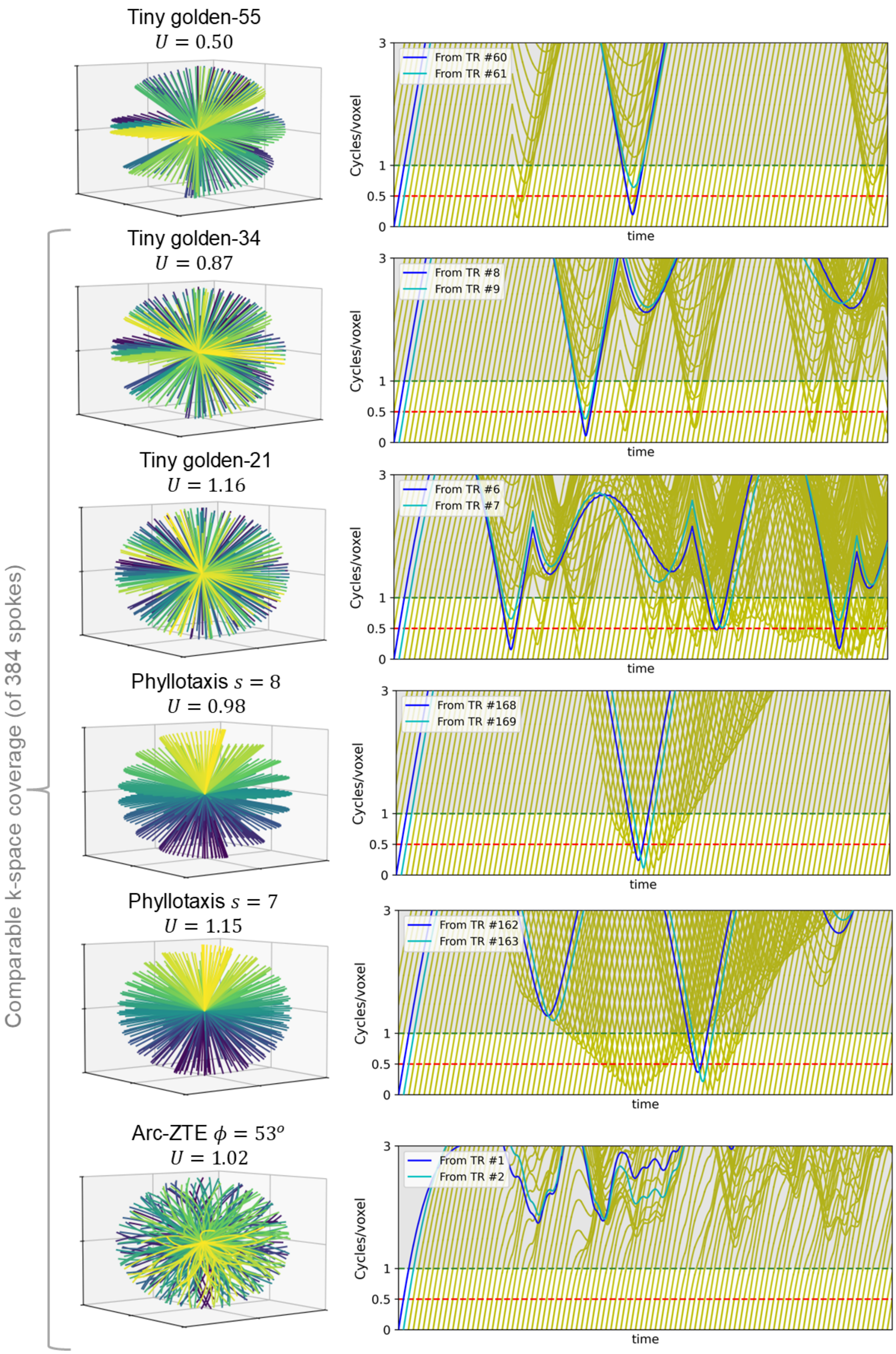

Figure S3: Trajectories and coverage uniformity U of 1 segment of 384 spokes, along with magnetic coherence pathways of 100 TRs, across interleaved view-ordering schemes. Color of the trajectory spokes indicate time of acquisition. Although tiny golden angle schemes can achieve comparable coverage uniformity to Arc-ZTE within 384 spokes, they result in several instances of gradient refocusing. Similarly, phyllotaxis schemes with slew rates higher than that used in the main manuscript can also achieve comparable coverage uniformity, but also result in instances of gradient refocusing.

4 Point spread functions of fully-sampled trajectories

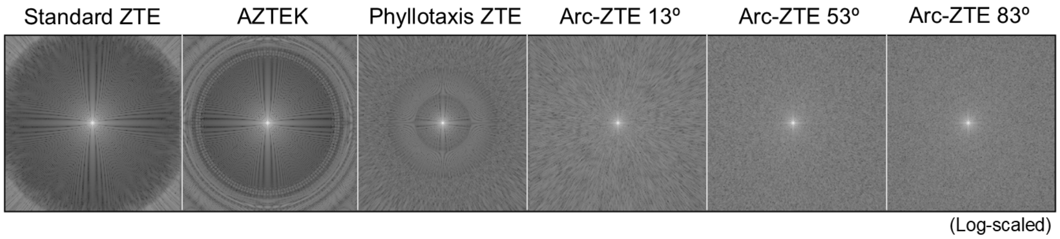

Figure S4: Comparison of a 2D cross-section from the 3D point spread functions of fully-sampled ZTE trajectories. Here, the full trajectory consists of 66048 spokes, which is the empirical Nyquist rate for a matrix size of 256, and corresponded to a scan time of around 2 min and 47 s. The Arc-ZTE PSFs continue to appear noise-like, due to the sampling incoherence throughout the complete trajectory.

5 Gradient roll-off artifacts observed in in-vivo ZTE

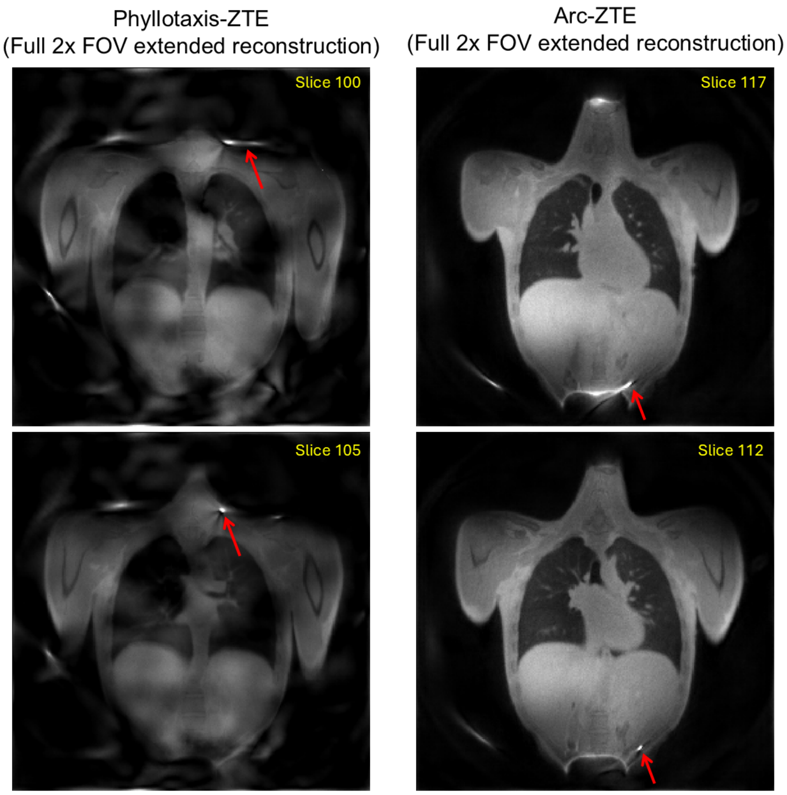

Figure S5: Example slices demonstrating bright artifacts near the edges of the extended FOV. Slices were taken from time-resolved reconstruction from Case 2 for both ZTE schemes. These artifacts are hypothesized to arise from the gradient roll-off, which collapses out-of-FOV signal into bright lines. In certain slices, this signal collapse appears as bright points, which appear larger in the phyllotaxis reconstruction due to the lower image quality. This signal can be attributed to both the surrounding tissue and plastic, due to the non-selective excitation and the zero echo time.

6 Analysis of respiratory motion in subspace-constrained reconstruction for DCE experiment

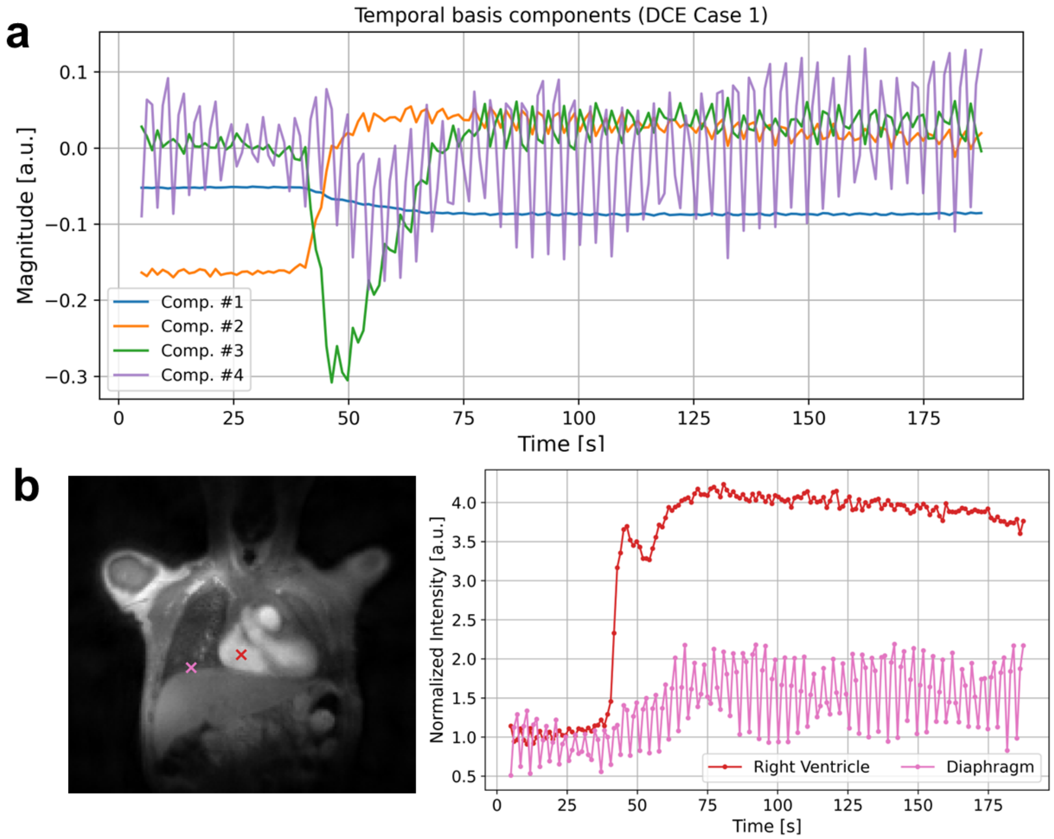

Figure S6: Demonstration of respiratory motion in DCE experiment reconstructed at 1.1s/frame. (a) Plot of the 4 most significant temporal basis components, which were extracted from an SVD of the low-resolution reconstruction. Components 1, 2,and 3 seem to capture the baseline and general contrast enhancement dynamics, while Component 4 appears to capture the respiratory motion. (b) Time-curves of two ROIs from the reconstruction: one from the diaphragm region and one from the right ventricle. The explicit low-rank constraint resolves the respiratory motion in regions where it is significant, such as the diaphragm, and filters out the motion elsewhere, such as the left ventricle.

7 Calibrating gradient delays

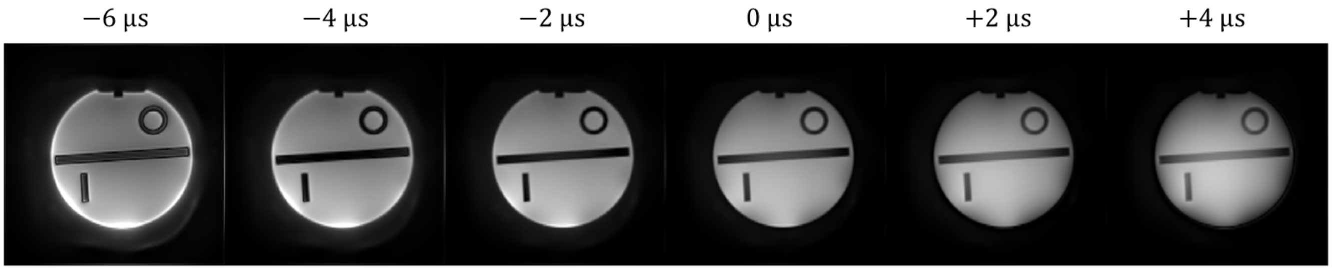

Figure S7: Empirical procedure to tune the timing offsets between the DAQ and gradients, by interpolating sampling coordinates along a fixed trajectory. A negative timing offset here indicates the gradient waveforms are delayed with respect to the DAQ (data acquisition), while a positive offset indicates the converse. Based on these images, a $-2\mu s$ offset of the gradients was used for this system. It should be noted that tuning the timing offset between the RF and gradients would instead involve generating new trajectory coordinates.
